# Assessment of osteoporosis using the FRAX method and the importance of vitamin D levels in COPD patients

**DOI:** 10.1186/s40248-017-0116-1

**Published:** 2018-01-06

**Authors:** Ceyda Anar, Melike Yüksel Yavuz, Filiz Güldaval, Yelda Varol, Dilek Kalenci

**Affiliations:** 1 Department of Chest Diseases, İzmir Dr. Suat Seren Chest Diseases and Surgery Training Research Hospital, Gaziler Cad. No: 331, 35110 İzmir, Turkey; 2 Department of Biochemistry, İzmir Dr. Suat Seren Chest Diseases and Surgery Training Research Hospital, İzmir, Turkey

## Abstract

**Background:**

The aim of this paper was to evaluate the availability of FRAX for assessing osteoporosis risk, and to demonstrate the importance of vitamin D levels in COPD patients.

**Methods:**

Fourty-six males who fulfilled the COPD diagnostic criteria defined by GOLD were included. Age, race, BMI, physical activity frequency, smoking and dietary habits, age at COPD diagnosis, disease duration, fractures history, and medications use were determined. Levels of 25(OH)D were detected. BMD was measured by DXA at lumbar spine, femoral neck, and entire femur, and classified according to ISCD. FRAX score was calculated. Control group was composed of 40 non-smoker individuals without previous history of pulmonary diseases.

**Results:**

25(OH)D levels were significantly different between patients and controls. In the COPD group, a statistically significant difference in vitamin D levels was detected among the A, B, C, and D grades, while no such significant differences in FRAX scores were detected. 25(OH)D levels were significantly low in COPD patients with disease exacerbations and hospitalizations in the previous one year. No correlation was detected between vitamin D levels and the FRAX score. A positive correlation was observed between vitamin D levels and T-score. FRAX scores were higher and vitamin D levels were lower in osteoporotic COPD patients than in non-osteoporotic COPD patients.

**Conclusion:**

Using FRAX for assessing osteoporosis in COPD can reduce fracture risk and allow adequate treatment. Since vitamin D levels are related to exacerbations and hospitalizations, vitamin D supplementation may be needed in COPD patients, especially in those with high FRAX scores.

## Background

Chronic Obstructive Pulmonary Disease (COPD) is a major cause of chronic morbidity and mortality worldwide. In addition to progressive loss of lung function, extra-pulmonary comorbidities include osteoporosis, cardiovascular disease, and low skeletal muscle mass and function, all of which adversely affect health outcomes [1].

Osteoporosis is a systemic skeletal disorder that is characterized by compromised bone strength due to decreased bone density, leading to increased fracture risk; and bone mineral density (BMD) is a very useful estimate of fracture risk [2]. Several factors like systemic inflammation, use of oral and inhaled corticosteroids, and vitamin D deficiency have been suggested to interact with pathways of bone remodeling in patients with COPD [3]. BMD is diagnostic for osteoporosis and can accurately be measured using dual energy x-ray absorptiometry (DXA). According to the World Health Organization (WHO), T scores >−1 are accepted as normal, T scores between −1 and −2.5 are considered as osteopenia, and T scores of <−2.5 are defined as osteoporosis. Further, the WHO has developed (Fracture assessment (FRAX®) method, a web-based algorithm table for calculating 10-year risk of hip fracture or a major osteoporotic fracture based on individual case models that uses clinical risk factors and femur neck BMD values or T-scores [4–6].

Currently, vitamin D is thought to play an important role in COPD and related systemic effects [7]. Additionally, in patients with advanced pulmonary diseases waiting for a lung transplants, vitamin D deficiency is associated with reduced femur neck T-scores [8]. Vitamin D deficiency not only leads to osteoporosis, but is also related to more frequent disease exacerbations and hospitalization in the previous 1 year [9].

Therefore, the aim of this study was to evaluate osteoporosis risk in COPD patients using FRAX and compare FRAX results with T scores obtained from DXA. We also investigated other factors that affect bone mass, such vitamin D levels and used this data to estimate the level of vitamin D that is associated with severe airway obstruction, disease exacerbation, and hospital admission in the previous 1 year.

## Methods

### Patients

The study protocol was approved by the hospital ethics committee (Dr. Suat Seren Chest Diseases and Surgery Training Hospital). Informed written consent was obtained from all subjects. We consecutively recruited 46 male patients (mean age 61.3 ± 1.2 years) from the Dr. Suat Seren Training and Research Hospital, Turkey.

We included only males who fulfilled the COPD diagnostic criteria defined by the Global Initiative for Chronic Obstructive Lung Disease (GOLD) [10]. The exclusion criteria applied were: prolonged immobilization within the past 6 months, presence of comorbidities or use of drugs that interfere with bone metabolism, and oral or intravenous glucocorticoids use for three or more consecutive months.

### Methods

Participants were evaluated during a single visit that included an interview, physical examination, laboratory testing, BMD measurement, and spirometry. Medical history was obtained through a questionnaire administered by a single investigator or from the patient charts and included information such as age, race, body mass index (BMI), frequency of physical activity, smoking and dietary habits, age at COPD diagnosis, duration of disease (years), history of fractures, and use of medications (past and present). Present and past smoking histories, as well as smoking intensity, expressed as number of cigarette packs per day per year of smoking, were also determined.

Patients who had stopped smoking less than 6 months prior to the evaluation were considered as present smokers. Calcium intake was estimated by the amount of dairy products ingested daily.

Levels of 25(OH)D were determined by a radioimmunoassay (I125 DiaSorin Stillwater, Minnesota, USA). BMD was measured at the lumbar spine, femoral neck, and the entire femur by DXA using a Hologic 1000 densitometer (Hologic, Bedford, MA, USA). BMD was classified as normal, low, or osteoporosis according to the International Society for Clinical Densitometry [11], and FRAX score was calculated.

A non-smoker group of 40 healthy individuals matched by age, gender, ethnicity, and BMI, without previous history of pulmonary diseases was used as controls and for comparison. All COPD patients were given pulmonary function tests, namely forced expiratory volume in 1 s (FEV_1_), the forced vital capacity (FVC), and the FEV_1_/FVC ratio; patients with FEV_1_/FVC < 0.7 were diagnosed with COPD [10].

### Statistical analysis

Data were analyzed using the SPSS 13.0 for Windows (SPSS, Chicago, IL, USA). Results are expressed as mean ± SD or median (range). Group means were compared using Student’s t-test or by non-parametric tests, as applicable. One-way analysis of variance (ANOVA) or chi-square test was used, as appropriate. Pearson’s and Spearman’s coefficients were used for correlation analysis. All analyses used two-sided tests and *p* < 0.05 was considered significant.

## Results

**Fig. 1:**
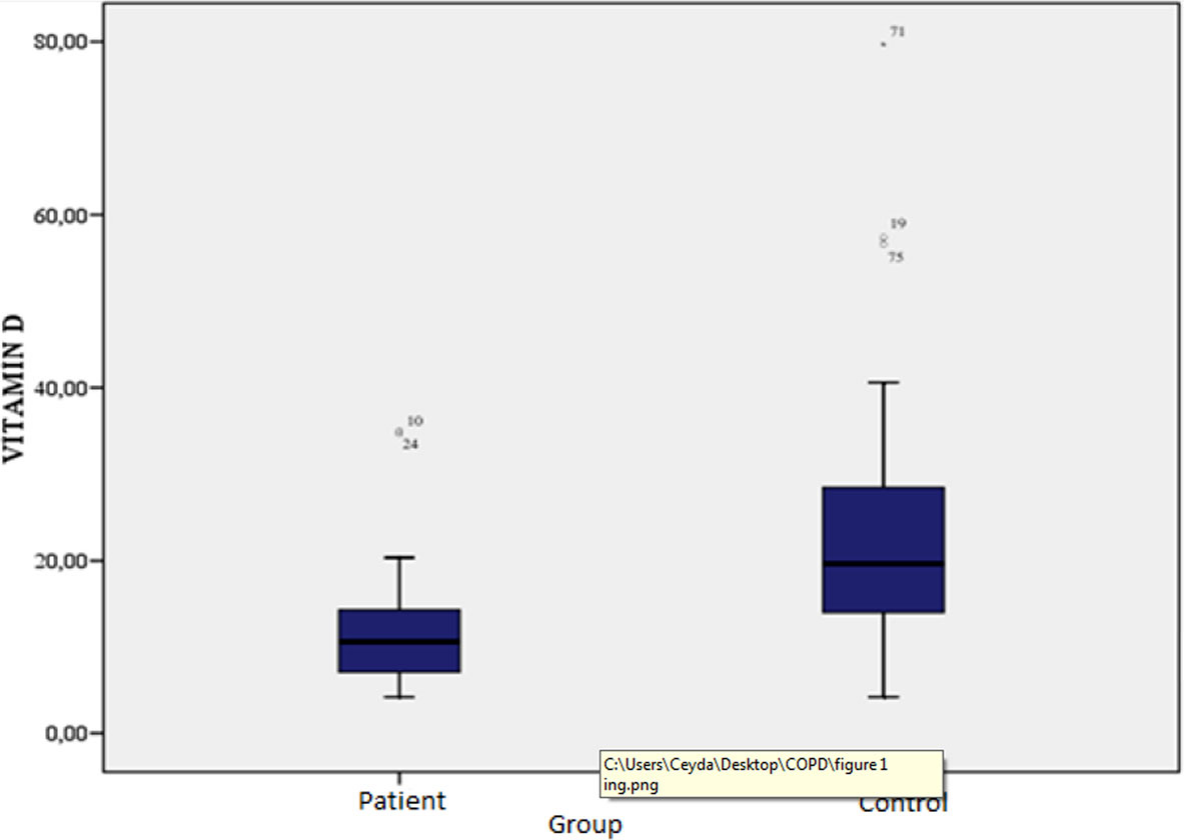
Comparison of 25(OH)D vitamin levels between patient group and control group

**Fig. 2:**
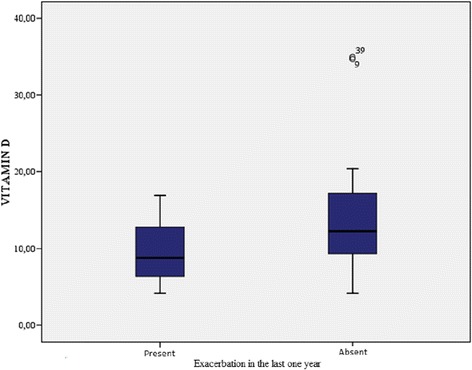
Relation between the exacerbation in the last year and vitamin D level

**Fig. 3:**
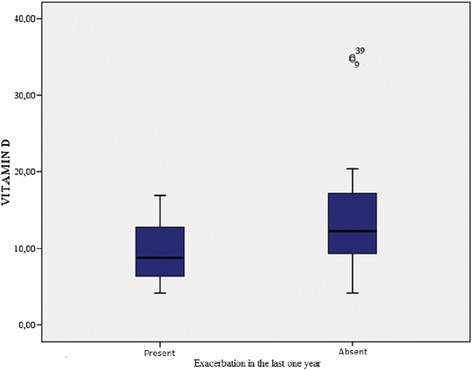
Relation between the hospitalization in the last year and vitamin D level

**Fig. 4:**
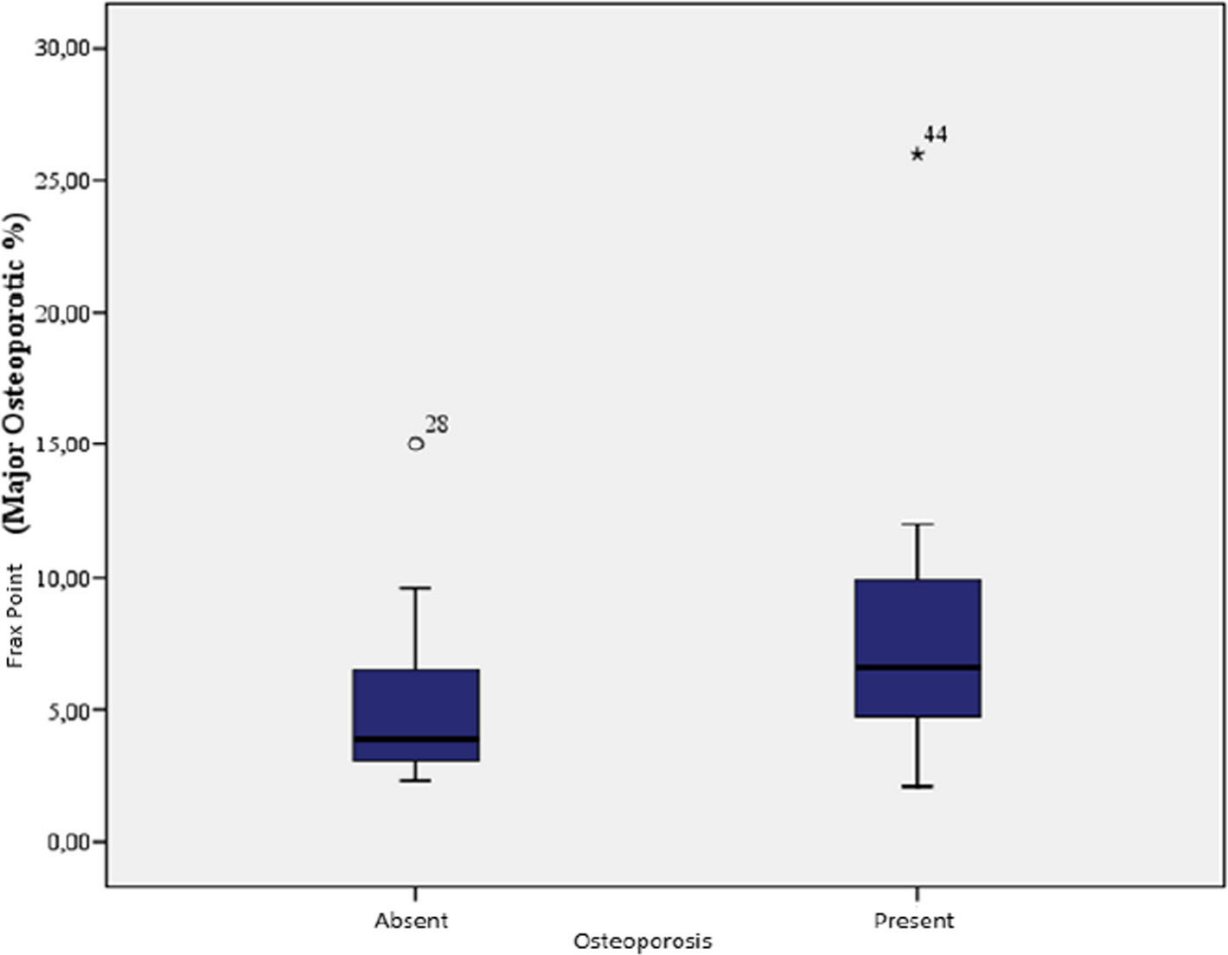
Relation between osteoporosis and FRAX score (major osteoporotic %)

**Fig. 5:**
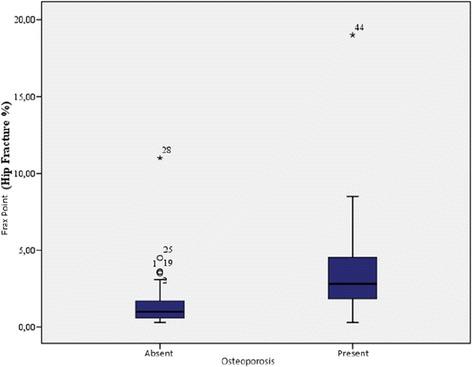
Relation between osteoporosis and FRAX score (hip fracture %)

**Fig. 6:**
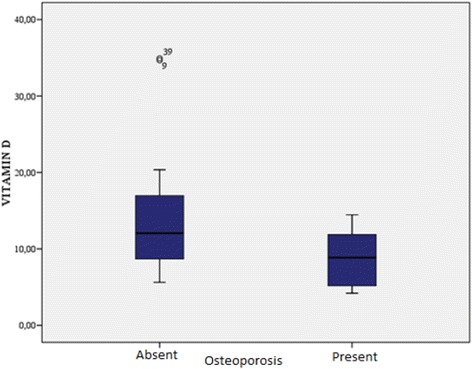
Relation between osteoporosis and vitamin D level

The demographic characteristics, laboratory results, and pulmonary function tests of patients with chronic obstructive pulmonary disease are shown in Table 1. The mean age of the COPD group was 61.3 ± 1.2 y and that of the control group was 57.1 ± 10.6 y. The GOLD grading system was used to further categorize COPD patients out of whom 30.4% were grade A, 15.2% grade B, 15.2% grade C, and 39.1% grade D. The levels of 25(OH)D were significantly different between patients and controls (*p* = 0.000; Fig. 1). In the COPD group, a statistically significant difference in vitamin D levels (*p* = 0.000) was detected among the A, B, C, and D grades, while no significant differences in FRAX scores (major osteoporosis and hip fracture) were detected (Table 2). Furthermore, levels of 25(OH)D were significantly lower in COPD patients with disease exacerbations and hospitalizations in the previous year than in COPD patients without exacerbations or hospitalizations (*p* = 0.014, 0.003, respectively; Figs. 2 and 3). Correlation analyses revealed a positive correlation between FEV_1_ values and 25(OH)D levels (*r* = 0.564, *p* = 0.000) and a negative correlation between COPD assessment test (CAT) score and 25(OH)D levels (*r* = −0.350, *p* = 0.02). Although no correlation was detected between vitamin D and the FRAX score, a positive correlation between T-score and vitamin D levels was observed (Table 3). In COPD patients using inhaled steroids, FRAX scores were numerically higher and vitamin D levels were lower compared to the controls, but these differences were not statistically significant. COPD patients with T-score <−2.5 were diagnosed as having osteoporosis and FRAX scores (major osteoporotic and hip fracture, *p* = 0.027 and 0.009, respectively) were higher (Figs. 4 and 5) and vitamin D levels were lower (*p* = 0.013) in osteoporotic COPD patients than in non-osteoporotic COPD patients (Fig. 6). Although no statistically significant differences were detected between osteoporosis development and smoking, comorbidity, COPD grade, hospitalization, inhaled steroid use, a significant difference was detected between osteoporosis development and COPD exacerbation in the last year (*p* = 0.036).

**Table 1:** Characteristics of COPD group

Feature	Mean ± SD	Median (min–max)
Age	62.4 ± 8.3	65 (41–76)
Smoking (PY)	43.8 ± 15.7	40 (20–100)
COPD time from diagnosis	7.6 ± 5.8	5 (2–25)
FEV_1_(mL)	1435 ± 597	1285 (400–2720)
FVC (mL)	2516 ± 777	2360 (840–4000)
FEV_1_/FVC (%)	55.8 ± 9.1	57 (33–70)
BMI (kg/m^2^)	24.6 ± 4.8	24 (14–38.6)
MRC	1.2 ± 0.6	2.2 (0–3)
CAD	11.5 ± 4.5	11 (4–26)
Exacerbation number	0.65 ± 0.9	0 (0–4)
Hospitalization number	0.24 ± 0.4	0 (0–2)
Vitamin D	12 ± 6.6	10.9 (4.2–34.9)
FRAX score (major osteoporotic)	6.09 ± 4.2	4.7 (2.1–26)
FRAX score (hip fracture)	2.5 ± 3.3	1.6 (0.3–19)
Lumber T Score	−1.4 ± 1.5	−1.6 (−4.3–2.7)
Neck T Score	−1.6 ± 0.9	−1.7 (−4–0.6)

**Table 2:** Characteristics of patients as per the COPD grades and Vitamin D levels

Feature	COPD grade		*p*
	A–B	C–D	
Age	65 (41–76)	65 (52–79)	0.320
Smoking (PY)	40 (20–80)	50 (20–100)	0.250
COPD time from diagnosis	4 (2–29)	5 (2–20)	0.178
FEV_1_/FVC (%)	64 (48–70)	51 (33–64)	0.000
FEV_1_ (mL)	66 (44–80)	35 (14–67)	0.000
FVC (mL)	86 (61–100)	54 (23–86)	0.000
BMI	25 (14–32)	23 (19–38.6)	0.956
MRC	1 (0–2)	2,2 (0–3)	0.011
CAT	9 (4–19)	12 (6–26)	0.012
Vitamin D	14.7 (7–34.9)	8.6 (4.2–16.9)	0.000
FRAX score (major osteoporotic %)	4.4 (2.3–15)	4.7 (2.1–26)	0.956
FRAX score (hip fracture %)	1.4 (0.3–11)	1.6 (0.3–19)	0.740

**Table 3:** Relation between Vitamin D level and FRAX score, MRC, and CAT situation

	Vitamin D	
FEV_1_/FVC (%)	*r*	0.436
	*p*	0.003
FEV _1_ (ml)	*r*	0.564
	*p*	0.000
FVC (ml)	*r*	0.514
	*p*	0.000
MRC	*r*	−0.464
	*p*	0.002
CAT	*r*	−0.350
	*p*	0.022
FRAX score (major osteoporotic %)	*r*	−0.090
	*p*	0.565
FRAX score (hip fracture %)	*r*	−0.170
	*p*	0.276
Lumbar spine T score	*r*	0.366
	*p*	0.016
Neck T score	*r*	0.422
	*p*	0.005

## Discussion

To the best of our knowledge, this is the first study in male COPD patients on quantifying osteoporosis risk based on both DXA scan and FRAX scores. The FRAX score was found to be significantly higher in patients with osteoporosis and was negatively correlated with vitamin D levels. While our data show no relation between inhaled steroid use and FRAX score, we detected that vitamin D levels were significantly lower in male COPD patients compared to healthy males and that low vitamin D levels were related to COPD exacerbations. Similar results have been previously reported [9, 12].

In our study, the mean FRAX score for major osteoporosis in the COPD group was higher than that of age- and gender- matched control subjects. Also, FRAX major osteoporotic and hip fracture percents in patients with osteoporosis according to bone mineral density were higher and this difference was statistically significant.

Studies in the general population have shown a correlation between FEV_1_ and BMD, suggesting that impaired pulmonary function may affect bone health [13–15]. Similarly, we found a positive correlation between FEV_1_ and T-scores at all sites evaluated. Nonetheless, other studies report divergent findings [16–18], possibly due to differences in subjects and methodologies.

We report that previous use of inhalatory glucocorticoids is not associated with changes in BMD or reduction in T-scores; previous studies both concur [19–21] and differ with our findings [22].

While some studies have shown that low vitamin D levels are associated with increased frequency of respiratory infections in both COPD patients and healthy adults [23, 24], however, the role of vitamin D deficiency in acute exacerbation of chronic obstructive pulmonary disease (AECOPD) is still debated. A recent study in exacerbation-prone COPD patients found no association between baseline vitamin D levels and subsequent risk of AECOPD [12]. However, according to Heulens et al. [25], this negative finding might have been influenced by the fact that some of the patients with worse clinical conditions were taking vitamin D supplements, and supplementation would obviously negate the effects of the underlying deficiency on COPD severity. In fact, by excluding data on those taking supplements during analyses, Heulens et al. [25] demonstrated that patients with vitamin D levels below 10 ng/mL had the shortest time to first exacerbation and that they experienced the highest number of AECOPD events. However, another recent study by Puhan et al. [26] reports no relationship between severe vitamin D deficiency and exacerbations and no effect of vitamin D supplementation on AECOPD. Thus, the benefits of vitamin D supplementation in COPD are still being debated. High dose vitamin D supplementation has been demonstrated to decrease number of AECOPD events but only in patients with severe deficiency [27] and only to improve inspiratory muscle strength and maximize oxygen uptake [28]. The results from our study show that both exacerbation and hospitalization frequency in the last year was low for COPD patients with low vitamin D levels.

We also show a positive correlation between FEV_1_ values and 25(OH)D levels and a negative correlation between CAT score and 25(OH)D levels. Furthermore, even though no correlation between vitamin D and FRAX scores was detected, a positive correlation between T-score and vitamin D was obtained. **So, the role of FRAX (major osteoporosis and hip fracture) has had a minor role compared to BMD in this study.** However, according to the new GOLD classification, vitamin D levels in COPD patients in our study was estimated to be significantly lower in grade C and D patients compared to grade A and B patients. In support of these findings, it has been reported that in a general population, levels of vitamin D and FEV_1_ are strongly correlated [29].

In conclusion, our findings clearly show that COPD patients, without chronic use of systemic glucocorticoids, are at increased risk for osteoporosis and low levels of vitamin D and that this is correlated with the disease severity. Inhalatory glucocorticoid use in a sub-sample of the study population was not associated with changes in T scores or FRAX scores. Moreover, as FRAX may be an easy method for assessing osteoporosis in a systemic disease like COPD, our results suggest that patients with COPD should routinely have their BMD evaluated by DXA or FRAX, even in the absence of severe disease or glucocorticoid use. This approach can reduce risk of fracture and could allow adequate treatment of osteoporosis. As we also show that vitamin D levels were lower in osteoporotic patients and that low vitamin D levels are related to number of exacerbations and hospitalizations in the last year, vitamin D supplementation may be needed in all patients with COPD, and especially in those with high FRAX scores. **Moreover, vitamin D supplementation significantly reduces the episodes of exacerbation and, therefore, hospitalization, thus influecing favourably the costs of pulmonary disease.** Nevertheless, future studies are needed to evaluate the relationship between vitamin D supplementation and the long-term risk of fractures in patients with COPD.

## Conclusion

Using FRAX for assessing osteoporosis in COPD can reduce fracture risk and allow adequate treatment. Since vitamin D levels are related to exacerbations and hospitalizations, vitamin D supplementation may be needed in COPD patients, especially in those with high FRAX scores.
